# Lack of Social Support and Its Role on Self-Perceived Health in a Representative Sample of Spanish Adults. Another Aspect of Gender Inequality

**DOI:** 10.3390/jcm10071502

**Published:** 2021-04-04

**Authors:** Julia Wärnberg, Napoleón Pérez-Farinós, María Julia Ajejas-Bazán, Jéssica Pérez-López, Juan Carlos Benavente-Marín, Edelys Crespo-Oliva, Virginia Castillo-Antúnez, Olga Fernández-Barceló, Silvia Valenzuela-Guerrero, María Ángeles Silva-Soto, F. Javier Barón-López

**Affiliations:** 1 Epi-PHAAN Research Group, School of Health Sciences, Universidad de Málaga, Instituto de Investigación Biomédica de Málaga (IBIMA), 29071 Málaga, Spain; jwarnberg@uma.es (J.W.); jessicaperezlopez@uma.es (J.P.-L.); jc.benaventemarin@gmail.com (J.C.B.-M.); edelys@uma.es (E.C.-O.); virginia.castillo.antunez@gmail.com (V.C.-A.); olga.fb@hotmail.com (O.F.-B.); svalgue@outlook.com (S.V.-G.); angelessilva_@hotmail.com (M.Á.S.-S.); baron@uma.es (F.J.B.-L.); 2 Centro de Investigación Biomédica en Red Fisiopatología de la Obesidad y la Nutrición (CIBEROBN), Institute of Health Carlos III, 28029 Madrid, Spain; 3 Epi-PHAAN Research Group, School of Medicine, Universidad de Málaga, Instituto de Investigación Biomédica de Málaga (IBIMA), 29071 Málaga, Spain; 4 Faculty of Nursing, Physiotherapy and Podiatry, Complutense University, 28040 Madrid, Spain; majejas@ucm.es

**Keywords:** self-perceived health, health status, social support, gender inequality

## Abstract

Self-perceived health has been used as a good estimator of health status and receiving affection can be a determining factor for good self-perceived health. The aim of the present study was to assess whether lack of social support (measured through Duke scale, which ranges from 11 to 55) was associated with poorer health status measured as self-perceived health, and whether that association was different between women and men. A cross-sectional study was conducted using data from the 2017 Spanish National Health Survey. A descriptive study was performed, and logistic regression models were applied using self-perceived health as a dependent variable. Mean Duke score was 47.6 for men and 47.9 for women (*p* = 0.016). Moreover, 36.3% of women and 27.6% of men reported poor self-perceived health (*p* < 0.001). The multivariate analysis revealed that lower scores in Duke-UNC social support scale were associated with poorer health status. That association was higher in women than in men. Poor self-perceived health was also associated with low level of education and obesity, especially among women. There was gender inequality as regards health status associated with lack of social support. These results can help design prevention strategies to improve health.

## 1. Introduction

Self-perceived health has been used for the past few years as a reliable indicator of several aspects related with health and, in particular, as a good estimator of health status [1,2,3]. It has also been considered a good morbidity predictor in people with chronic diseases and a good mortality predictor in adults, as well as a predictor of health care utilisation, especially among the elderly [4,5,6,7,8,9,10]. Furthermore, a high correlation has been observed between self-perceived health and health status assessed by health professionals [11]. Apart from physical aspects, there are several factors that affect self-perceived health, such as behavioural, social, or psychological factors [12,13,14,15].

Apart from the classical factors that determine health, social support has been described to have relevant influence on health and mortality [16,17,18]. Moreover, receiving affection can represent a determining factor for good self-perceived health [19], while the lack of social support can be very relevant in some population groups, like the elderly [20,21].

Attachment processes may be relevant to understanding how adult relationships can influence health and wellbeing [22]. The attachment theory originally proposed by Bowly in 1969 involved infant–caregiver bonds, but it currently plays an important role in how relationships are maintained throughout the lifespan. Attachment has been linked to health processes and is closely related to how people regulate affection, particularly when they feel threatened [23,24]. Parental style is a psychological construct that includes strategies to rear children, and it is supposed to be related to future behaviours in adolescent and adult life. The type of parental style has been suggested to play a role in social support [25].

The existence of inequalities among different population groups constitutes an aggravating factor to problems derived from health itself. Thus, the most disadvantaged groups experience more difficulties in maintaining good health status. Gender inequalities have been documented in numerous fields [26,27,28]. Gender health inequalities are defined as a systematic gap in health between men and women [29]. Women have been shown to be at a disadvantage as regards most health, social, and environmental determinants [30]. Gender differences in health have been associated with men’s and women’s social positions in contemporary society. Research on men’s health has been dominated by a structural framework focusing on occupational class differentials in morbidity and mortality, whereas women’s health has been studied primarily using a role framework, emphasising women’s roles as housewives and mothers, with paid employment seen as an additional role [31,32].

Women have higher life expectancy than men in more developed countries, but women suffer higher disease burden than men. This is called the ‘gender paradox’, and it means that a longer life does not involve better health [33]. Women suffer more frequent non-mortal chronic diseases with long-term disability, while men suffer more potentially-mortal diseases with permanent disability [34].

These gender health inequalities also seem to exist in health status, many of them due to social or economic factors [35].

Given the above, it seems interesting to determine whether the lack of social support has influence on health status, measured as self-perceived health. This would represent a step forward regarding health data collection, as well as a new approach as regards prevention in health.

The aim of the present study was to assess whether the lack of social support was associated with poorer health status measured as self-perceived health, and whether that association differed between women and men.

## 2. Materials and Methods

### 2.1. Design, Setting and Participants

Individual secondary data from the 2017 Spanish National Health Survey (SNHS) were used to conduct a cross-sectional study. The SNHS is an interview-based survey conducted by the Ministry of Health in collaboration with the National Statistics Institute (Instituto Nacional de Estadística) that includes a large sample of Spanish non-institutionalised population. The survey used multistage cluster sampling, with proportional random selection of primary and secondary sampling units (towns and sections, respectively). The final units (individuals) were selected by means of random routes and sex- and age-based quotas. The 2017 survey included 23,089 adults of both sexes interviewed between October 2016 and October 2017. Details of SNHS methodology are described elsewhere [36]. The present study included 20,233 people (9415 men and 10,818 women) aged 18 to 84 whose information for all variables under study was available.

### 2.2. Main Outcome Measures

The independent variable recorded whether an individual received social support. The SNHS contains the 11 items of Duke-UNC social support score: ‘I receive visits from friends and relatives’, ‘I receive help around the house’, ‘I receive praise for a good job’, ‘I have people who care what happens to me’, ‘I get love and affection’, ‘I get chances to talk to someone about problems at work or with my housework’, ‘I get chances to talk to someone about my personal or family problems’, ‘I get chances to talk to someone about money matters’, ‘I get invitations to go out and do things with other people’, ‘I get useful advice about important things in life’, ‘I get help when I am sick in bed’. Each item has five possible answers: ‘Much less than I would like’, ‘Less than I would like’, ‘Not much, not little’, ‘Almost as much as I would like’ and ‘As much as I would like’, in a Likert-type scale from 1 to 5 points. Duke scale ranges from 11 to 55. Duke-UNC social support scale was developed by Broadhead et al. in 1988 [37] and it was adapted to Spanish by Bellón et al. in 1996 [38].

Self-perceived health was used as a dependent variable. The SNHS establishes five categories (Very good, Good, Fair, Poor and Very poor). The answers were recoded into two categories: Good (including Very good and Good) and Poor (including Fair, Poor and Very poor).

Age, cohabitation with a partner, level of education, Spanish nationality, obesity, chronic health problems, depression and cardiovascular disease were added as covariates. The age was coded into three categories: 18 to 44 years, 45 to 64 years and 65 to 84 years.

The level of education was coded into three categories: No studies or primary studies, Secondary studies and University studies. Obesity was defined as a body mass index (BMI) value equal to or higher than 30 kg/m^2^, calculated from self-reported weight and height values.

The variables chronic disease, depression and cardiovascular disease were collected in order to control for the effect of suffering from a chronic disease on self-perceived health. To do so, participants were asked whether they had suffered from any type of chronic disease, depression or cardiovascular disease (Yes/No).

### 2.3. Statistical Analysis

A descriptive analysis of the sample was conducted based on the dependent and independent variables, as well as the covariates. The differences between men and women were assessed for all variables. The differences in all variables were also assessed for men and women in every level of education. Duke score differences were examined using Student’s *t*-test for independent samples (between sexes) and chi-squared test for the rest of variables. Differences between sexes within every age group and level of education were assessed using comparison of proportions (with Bonferroni-corrected *p*-values).

Logistic regression models were applied to determine the independent effect of receiving social support on self-perceived health. Self-perceived health was the dependent variable.

In the first model the estimates were assessed separately for men and women and for every age group. In the second model the estimates were assessed separately for men and women and for every level of education. Duke-UNC score was the independent variable, and level of education or age group (depending on the model) chronic health problems, obesity, depression and cardiovascular disease were included as covariates. The variable presence of chronic disease was also added.

IBM SPSS Statistics 25.0 (IBM Corp. Released 2017. IBM SPSS Statistics for Windows, Version 25.0. Armonk, NY, USA: IBM Corp) was used for statistical analysis and significance was set at two-tailed <0.05.

### 2.4. Ethical Aspects

In accordance with Spanish law, the approval by an ethics committee was not needed as a public-access dataset with anonymous data was used. The database can be freely downloaded [39].

## 3. Results

Out of the 20,233 people included in 2017 SNHS, 53.5% were women (*n* = 10,818). Table 1 contains the sample description divided by sex.

The percentage of women with university studies was 28.1%, while the percentage of men was 27.5% (*p* < 0.001). Obesity was observed in 19.7% of men and 18.6% of women (*p* = 0.038). Mean Duke score was 47.8 (SD 7.32), 47.6 in men and 47.9 in women (*p* = 0.016). Besides, 72.4% of men and 63.7% of women perceived their health as good (*p* < 0.001).

The percentage of women with chronic health problems was significantly higher than the percentage of men in all levels of education. The highest percentage corresponded to women with no studies or with primary studies (Table 2). For the lowest level of education, the percentage of women with obesity was higher than the percentage of men. By contrast, for secondary and university education levels, the percentage of men with obesity was higher than the percentage of women.

The percentage of people who perceived their health as poor was significantly higher among those with the lowest level of education compared to those with secondary or university studies. In all levels of education, the percentage of women who presented poor self-perceived health was higher than the percentage of men.

The percentage of people who lived with a partner was significantly higher in the group who had completed university studies (66.0% of men and 56.7% of women with this education level). Percentages were higher for men than for women in all education levels.

The percentage of women who reported depression was approximately twice as much as that of men, the highest value corresponding to women with no studies or primary studies. The percentage of men with cardiovascular disease was significantly higher than the percentage of women, particularly in the lowest level of education.

The logistic regression model by age group and sex (Table 3) revealed that the risk of perceiving their health as poor was lower among people with high Duke score, the risk being lower in women than in men in all age groups. The risk of presenting poor self-perceived health was higher in women than in men with no studies or primary studies aged 45 to 64 and 65 to 84.

Chronic health problems were found to be associated with significantly higher risk of presenting poor self-perceived health in women aged 45 to 64 and 65 to 84 than in men. In the group aged 18 to 44, the risk was similar for men and women. Obesity was associated with significantly higher risk of presenting poor self-perceived health in women aged 18 to 44 and 65 to 84 than in men.

The logistic regression model by level of education and sex (Table 4) showed that the risk of perceiving their health as poor was lower among people with higher Duke score, the risk being higher in women than in men in all levels of education. The risk of presenting poor self-perceived health was significantly higher in women than in men with no studies or with primary education.

Chronic health problems were found to be associated with significantly higher risk of presenting poor self-perceived health in women than in men in all levels of education. Obesity was associated with significantly higher risk of presenting poor self-perceived health in women than in men with no studies or with primary studies, as well as with secondary studies.

Living with a partner and Spanish nationality were not significantly associated with self-perceived health.

## 4. Discussion

The main finding of this population-based study was that lower scores in the Duke-UNC social support scale were associated with poorer self-perceived health. Moreover, self-perceived health was poorer in women than in men with low social support in all age groups and all levels of education.

It is widely accepted that social and economic factors influence people’s health [40,41]. Among them, social support seems to have an effect on health [42]. In fact, the lack of relationships or social bonds was associated with physical or mental disorders [43,44,45,46,47]. Furthermore, social isolation, especially among the elderly, leads to lower health care utilisation, which produces poorer health status [48]. Perceived social support protects lonely people, and this has become more evident during the COVID-19 pandemic [49]. EUROASPIRE-7 study, conducted across 27 European countries, found that social isolation and low level of education were associated with various cardiovascular risk factors [50].

Thus, social isolation and lack of social support are becoming increasingly important factors affecting health, and social support has even been proposed as a potential public health intervention [51].

Our study revealed that a greater percentage of women than of men reported poor self-perceived health and chronic health problems. In principle, this could be the cause of such poorer health. However, the adjusted multivariate analysis results suggest that there could be other underlying causes. In addition, since the analysis controlled for some health problems (chronic diseases, obesity, depression and cardiovascular disease) which are related to self-perceived health, the inequalities found in self-perceived health can also be considered to be inequalities in health status.

Thus, the major finding of the present study was that the lack of social support was independently associated with poor health status, even after adjusting for several variables that affect health status, especially chronic disease, obesity, depression, cardiovascular disease or level of education. Furthermore, women who received less social support were more likely to present poor self-perceived health than men, revealing a gender inequality in health status.

In our study, self-perceived health was poorer in women than in men in almost all groups analysed, particularly in all levels of education. This could be explained by the fact that gender health inequalities are still present in our society. Women still play a different social role as regards family and work [52]. A recent literature review on remote working due to the pandemic conducted by Oakman et al. found that working at home reduced the opportunities for women to improve their health and was associated with poorer self-perceived health [53]. Besides, Chai et al. described that non-spousal social support was more important for women’s than men’s health protection [54]. This may also be related to the percentage of women with no studies or primary education, which is higher than that of men. The association between poor self-perceived health and low level of education, especially among women, has been previously described. A recent study conducted in Colombia by Mendoza-Romero et al. showed that low socioeconomic status, measured as level of education, was associated with poorer self-perceived health in women [55]. The findings of the present study with regard to obesity as a factor for poor self-perceived health, especially in women with low level of education, are in keeping with previous research [55,56,57].

The mechanisms influencing the relationship between affection reception and self-perceived health are not completely clear. There are theories that suggest that social support is associated with the modulation of the hypothalamus-hypophysis axis and with neurotransmitters of the autonomic nervous system, such as oxytocin and vasopressin. This would reduce anxiety and stress and increase the feeling of security, which would improve self-perceived health [22,58,59].

Besides neuroendocrinological hypotheses, from a psychological approach it has been suggested that receiving affection and social support and their amount have determining impacts on health protection through psychological factors, both at individual and collective levels. Holt-Lunstad defined this as the ‘ecological model’ and proposed public health interventions based on social support [22]. The Attachment theory could also play a relevant role in health behaviours and perceived health. Every attachment style can make expectations of the response of partners concerning social support be seen in a different way. This fact could play a mediator role between social support and self-perceived health [23,24]. This concern should be born in mind when interpreting our results.

The strengths of this population-based study included the use of standardised surveys and questionnaires, as well as data collection performed by well-trained interviewers using a large representative sample of Spanish adults (more than 20,000 people). Moreover, the study analysed socio-demographic variables that are not frequently assessed through standardised methods in such a large population. Besides, the analyses controlled for factors that affect self-perception. This allows us to assume that the inequalities found were more related to health status than to self-perceived health.

On the other hand, the study presents certain limitations. Firstly, the SNHS includes information on numerous chronic diseases that obviously affect self-perceived health. Although the effect of suffering from a chronic disease, depression and cardiovascular disease was considered and controlled for in the present study, potential bias due to this factor should not be disregarded. Secondly, the survey was self-administered, which could have led to under- or over-declaration, recall bias or social desirability bias. Thirdly, the final non-answering rate in 2017 SNHS was 27.8%. Therefore, the existence of non-answering bias should be considered [60]. Finally, due to the cross-sectional study design, reverse causality bias cannot be disregarded.

## 5. Conclusions

This study confirmed that receiving little social support was associated with poorer health status measured as self-perceived health. Additionally, poorer self-perceived health was associated with chronic health problems, obesity and low level of studies, especially in women. The study revealed gender inequalities, since the lack of social support seems to be more strongly associated with poor health among women. These results can help design prevention and public health strategies in order to improve health.

## Figures and Tables

**Table 1:** Description of the study variables. Adult men and women. 2017 Spanish National Health Survey.

	Total		Men		Women		*p*
Self-perceived health, *n* (%)							
Good	13,712	(67.8)	6821	(72.4)	6891	(63.7)	
Poor	6521	(32.2)	2594	(27.6)	3927	(36.3)	<0.001
Duke scale, mean (SD)	47.8	(7.32)	47.6	(7.42)	47.9	(7.23)	0.016
Age group, *n* (%)							
18 to 44 years	7134	(35.3)	3429	(36.4)	3705	(34.2)	
45 to 64 years	7598	(37.6)	3654	(38.8)	3944	(36.5)	
65 to 84 years	5501	(27.2)	2332	(24.8)	3169	(29.3)	<0.001
Level of education, *n* (%)							
Primary or no studies	5795	(28.6)	2465	(26.2)	3330	(30.8)	
Secondary studies	8806	(43.5)	4358	(46.3)	4448	(41.1)	
University studies	5632	(27.8)	2592	(27.5)	3040	(28.1)	<0.001
Living with a partner, *n* (%)							
No	8531	(42.2)	3527	(37.5)	5004	(46.3)	
Yes	11,702	(57.8)	5888	(62.5)	5814	(53.7)	<0.001
Spanish nationality, *n* (%)							
No	18,919	(93.5)	605	(6.4)	709	(6.6)	
Yes	1314	(6.5)	8810	(93.6)	10,109	(93.4)	0.713
Chronic health problems, *n* (%)							
No	6346	(31.4)	3329	(35.4)	3017	(27.9)	
Yes	13,887	(68.6)	6086	(64.6)	7801	(72.1)	<0.001
Obesity, *n* (%)							
No	16,369	(80.9)	7559	(80.3)	8810	(81.4)	
Yes	3864	(19.1)	1856	(19.7)	2008	(18.6)	0.038
Depression, *n* (%)							
No	16,883	(83.4)	8425	(89.5)	8458	(78.2)	
Yes	3350	(16.6)	990	(10.5)	2360	(21.8)	<0.001
Cardiovascular disease, *n* (%)							
No	18,386	(90.9)	8412	(89.3)	9974	(92.2)	
Yes	1847	(9.1)	1003	(10.7)	844	(7.8)	<0.001

*p*: *p*-value of statistical significance for the differences between sexes. SD: standard deviation.

**Table 2:** Description of the study variables depending on level of education and sex. Adult men and women. 2017 Spanish National Health Survey.

	Primary or No Studies					Secondary Studies					University Studies				
	Men		Women		*p*	Men		Women		*p*	Men		Women		*p*
Self-perceived health, *n* (%)															
Good Poor	1387	(56.3)	1432	(43.0)		3299	(75.7)	3000	(67.4)		2135	(82.4)	2459	(80.9)	
	1078	(43.7)	1898	(57.0)	<0.001	1059	(24.3)	1448	(32.6)	<0.001	457	(17.6)	581	(19.1)	0.158
Duke scale, mean (SD)	46.3	(8.0)	46.9	(7.6)	0.009	47.9	(7.5)	48.1	(7.3)	0.254	48.5	(6.5)	48.8	(6.5)	0.124
Age group, *n* (%)															
18 to 44 years 45 to 64 years 65 to 84 years	316	(12.8)	242	(7.3)		1905	(43.7)	1844	(41.5)		1208	(46.6)	1619	(53.3)	
	780	(31.6)	879	(26.4)		1861	(42.7)	1927	(43.3)		1013	(39.1)	1138	(37.4)	
	1369	(55.5)	2209	(66.3)	<0.001	592	(13.6)	677	(15.2)	0.031	371	(14.3)	283	(9.3)	<0.001
Living with a partner, *n* (%)															
No Yes	838	(34.0)	1674	(50.3)		1808	(41.5)	2015	(45.3)		881	(34.0)	1315	(43.3)	
	1627	(66.0)	1656	(49.7)	<0.001	2550	(58.5)	2433	(54.7)	<0.001	1711	(66.0)	1725	(56.7)	<0.001
Spanish nationality, *n* (%)															
No Yes	2312	(93.8)	3187	(95.7)		4042	(92.7)	4042	(90.9)		2456	(94.8)	2880	(94.7)	
	153	(6.2)	143	(4.3)	0.001	316	(7.3)	406	(9.1)	0.002	136	(5.2)	160	(5.3)	0.978
Chronic health problems, *n* (%)															
No Yes	462	(18.7)	373	(11.2)		1792	(41.1)	1433	(32.2)		1075	(41.5)	1211	(39.8)	
	2003	(81.3)	2957	(88.8)	<0.001	2,566	(58.9)	3015	(67.8)	<0.001	1517	(58.5)	1829	(60.2)	0.221
Obesity, *n* (%)															
No Yes	1839	(74.6)	2409	(72.3)		3518	(80.7)	3668	(82.5)		2202	(85.0)	2733	(89.9)	
	626	(25.4)	921	(27.7)	0.029	840	(19.3)	780	(17.5)	0.037	390	(15.0)	307	(10.1)	<0.001
Depression, *n* (%)															
No Yes	2084	(84.5)	2245	(67.4)		3915	(89.8)	3542	(79.6)		2426	(93.6)	2671	(87.9)	
	381	(15.5)	1085	(32.6)	<0.001	443	(10.2)	906	(20.4)	<0.001	166	(6.4)	369	(12.1)	<0.001
Cardiovascular disease, *n* (%)															
No Yes	1980	(80.3)	2797	(84.0)		4017	(92.2)	4223	(94.9)		2415	(93.2)	2954	(97.2)	
	485	(19.7)	533	(16.0)	<0.001	341	(7.8)	225	(5.1)	<0.001	177	(6.8)	86	(2.8)	<0.001

*p*: *p*-value of statistical significance for the differences between sexes. SD: standard deviation.

**Table 3:** Risk of presenting poor self-perceived health based on Duke functional social support scale by age group and sex. Adult men and women. 2017 Spanish National Health Survey.

	18 to 44 Years						45 to 64 Years					
	Men			Women			Men			Women		
	OR	(95% CI)	*p*	OR	(95% CI)	*p*	OR	(95% CI)	*p*	OR	(95% CI)	*p*
Duke scale	0.69444	(0.599, 0.805 )	<0.00111	0.63111	(0.556, 0.718)	<0.00111	0.8	(0.721, 0.889)	<0.00111	0.721	(0.651, 0.799)	<0.001
Level of education												
University studies	1			1			1			1		
Secondary studies	1.16	(0.91, 1.49)	0.227	1.61	(1.32, 1.97)	<0.001	1.7	(1.39, 2.08)	<0.001	1.6	(1.33, 1.92)	<0.001
Primary or no studies	1.69	(1.17, 2.43)	0.005	1.53	(1.05, 2.23)	0.026	1.98	(1.56, 2.51)	<0.001	2.01	(1.62, 2.50)	<0.001
Chronic health problems												
No	1			1			1			1		
Yes	6.27	(4.79, 8.20)	<0.001	6.18	(4.85, 7.87)	<0.001	8.44	(6.40, 11.11)	<0.001	13.65	(9.81, 19.00)	<0.001
Obesity												
No	1			1			1			1		
Yes	1.16	(0.87, 1.55)	0.315	1.46	(1.13, 1.88)	0.004	1.32	(1.10, 1.60)	0.004	1.29	(1.07, 1.56)	0.007
Depression												
No	1			1			1			1		
Yes	4.25	(3.13, 5.78)	<0.001	3.74	(2.97, 4.71)	<0.001	4.22	(3.35, 5.31)	<0.001	3.11	(2.62, 3.69)	<0.001
Cardiovascular disease												
No	1			1			1			1		
Yes	3.88	(2.21, 6.80)	<0.001	1.27	(0.72, 2.24)	0.412	2.94	(2.27, 3.81)	<0.001	2.91	(2.05, 4.13)	<0.001

OR: odds ratio for 10-point increase; CI: confidence interval.

**Table 4:** Risk of presenting poor self-perceived health based on Duke functional social support scale by level of education and sex. Adult men and women. 2017 Spanish National Health Survey.

	Primary or No Studies						Secondary Studies						University Studies					
	Men			Women			Men			Women			Men			Women		
	OR	(95% CI)	*p*	OR	(95% CI)	*p*	OR	(95% CI)	*p*	OR	(95% CI)	*p*	OR	(95% CI)	*p*	OR	(95% CI)	*p*
Duke scale	0.82	(0.729, 0.922)	0.001	0.731	(0.655, 0.817)	<0.001	0.738	(0.666, 0.817)	<0.001	0.677	(0.612, 0.749)	<0.001	0.688	(0.586, 0.808)	<0.001	0.654	(0.565, 0.756)	<0.001
Age group																		
18 to 44 years	1			1			1			1			1			1		
45 to 64 years	1.41	(0.99, 2.01)	0.055	1.62	(1.11, 2.36)	0.012	1.9	(1.58, 2.30)	<0.001	1.34	(1.14, 1.59)	<0.001	1.28	(0.98, 1.66)	0.066	1.42	(1.14, 1.76)	0.002
65 to 84 years	1.71	(1.21, 2.40)	0.002	2.4	(1.67, 3.44)	<0.001	1.81	(1.42, 2.30)	<0.001	1.57	(1.27, 1.95)	<0.001	1.67	(1.21, 2.32)	0.002	1.39	(1.00, 1.92)	0.048
Chronic health problems																		
No	1			1			1			1			1			1		
Yes	10.33	(6.84, 15.60)	<0.001	13.18	(8.56, 20.29)	<0.001	6.86	(5.40, 8.72)	<0.001	8.18	(6.41, 10.45)	<0.001	6.94	(4.87, 9.89)	<0.001	7.61	(5.47, 10.59)	<0.001
Obesity																		
Yes	1			1			1			1			1			1		
No	1.2	(0.98, 1.47)	0.086	1.24	(1.04, 1.48)	0.018	1.17	(0.96, 1.41)	0.112	1.61	(1.34, 1.93)	<0.001	1.37	(1.02, 1.83)	0.034	1.26	(0.93, 1.71)	0.133
Depression																		
Yes	1			1			1			1			1			1		
No	4.05	(3.10, 5.28)	<0.001	2.88	(2.41, 3.43)	<0.001	3.91	(3.12, 4.91)	<0.001	3.38	(2.85, 4.01)	<0.001	4.28	(3.00, 6.11)	<0.001	3.71	(2.90, 4.75)	<0.001
Cardiovascular disease																		
Yes	1			1			1			1			1			1		
No	2.8	(2.25, 3.57)	<0.001	2.77	(2.17, 3.54)	<0.001	2.37	(1.85, 3.05)	<0.001	2.68	(1.95, 3.68)	<0.001	3.23	(2.26, 4.61)	<0.001	1.34	(0.82, 2.17)	0.239

OR: odds ratio for 10-point increase; CI: confidence interval.

## Data Availability

This study was conducted using a public-access dataset. The database can be freely downloaded by anyone.
